# Orthotopic Transplantation of Achilles Tendon Allograft in Rats: With or without Incorporation of Autologous Mesenchymal Stem Cells

**DOI:** 10.1177/0963689717745891

**Published:** 2018-04-11

**Authors:** Michael Aynardi, Talal Zahoor, Reed Mitchell, Jeffrey Loube, Tyler Feltham, Lumanti Manandhar, Sharada Paudel, Lew Schon, Zijun Zhang

**Affiliations:** 1 Pennsylvania State University, Hershey, PA, USA; 2 Texas Tech University, Permian Basin, TX, USA; 3 MedStar Union Memorial Hospital, Baltimore, MD, USA; 4 The Johns Hopkins University, Baltimore, MD, USA

**Keywords:** Achilles tendon, transplantation, allograft, mesenchymal stem cells, rat

## Abstract

The biology and function of orthotopic transplantation of Achilles tendon allograft are unknown. Particularly, the revitalization of Achilles allograft is a clinical concern. Achilles allografts were harvested from donor rats and stored at −80 °C. Subcutaneous adipose tissue was harvested from the would-be allograft recipient rats for isolation of mesenchymal stem cells (MSCs). MSCs were cultured with growth differentiation factor-5 (GDF-5) and applied onto Achilles allografts on the day of transplantation. After the native Achilles tendon was resected from the left hind limb of the rats, Achilles allograft, with or without autologous MSCs, was implanted and sutured with calf muscles proximally and calcaneus distally. Animal gait was recorded presurgery and postsurgery weekly. The animals were sacrificed at week 4, and the transplanted Achilles allografts were collected for biomechanical testing and histology. The operated limbs had altered gait. By week 4, the paw print intensity, stance time, and duty cycle (percentage of the stance phase in a step cycle) of the reconstructed limbs were mostly recovered to the baselines recorded before surgery. Maximum load of failure was not different between Achilles allografts, with or without MSCs, and the native tendons. The Achilles allograft supplemented with MSCs had higher cellularity than the Achilles allograft without MSCs. Deposition of fine collagen (type III) fibers was active in Achilles allograft, with or without MSCs, but it was more evenly distributed in the allografts that were incubated with MSCs. In conclusion, orthotopically transplanted Achilles allograft healed with host tissues, regained strength, and largely restored Achilles function in 4 wk in rats. It is therefore a viable option for the reconstruction of a large Achilles tendon defect. Supplementation of MSCs improved repopulation of Achilles allograft, but large animal models, with long-term follow up and cell tracking, may be required to fully appreciate the functional benefits of MSCs.

## Introduction

Achilles tendon attaches the gastrocnemius and soleus muscles to the calcaneus and is essential for standing, walking, jumping, and other physical activities. Tendinopathy of Achilles tendon causes pain, foot dysfunction, and even tendon rupture^1^. It has been estimated that the likelihood of Achilles tendinopathy in a lifetime is about 10% for the general population and as high as 50% for male endurance runners^2,3^. There are many choices of treatments for Achilles tendinopathy, including physical therapy, rest, training modification, splintage, taping, cryotherapy, electrotherapy, shock wave therapy, hyperthermia, and local injections, but none of these therapies targets a specific pathology of tendinopathy^4^. For 25% to 40% of the patients with Achilles tendinopathy, these therapies are ineffective and surgical removal of the lesion becomes another option^5,6^. After surgery, some patients have persistent tendon structural abnormalities, which leave the recurrence of Achilles tendinopathy or rupture a possibility, for as long as 13 y^7^. An aggressive approach would be to eradicate the tendinopathic lesion or diseased tendon completely. However, it would also leave a large tendon defect for repair. There are other clinical circumstances where Achilles tendon is completely missing. For example, Achilles tendon has to be resected due to tumor tissue invasion^8,9^. In rare trauma cases, the entire, or a large portion of, Achilles tendon is destroyed^10,11^. When a large defect of Achilles tendon is left by either trauma or severe tendinopathy, it is a challenge to restore the continuity and function of the Achilles tendon, the largest tendon in the human body. An allograft of Achilles tendon would satisfy the required size and strength and does not have the supply limitation that autografts have.

As an allograft, Achilles tendon has been used to reconstruct joints^12,13^ and to repair rotator cuff^14^, patellar tendon^15^, and anterior cruciate ligament^16^, but only occasionally transplanted to replace an Achilles tendon. In a group of 14 cases, an Achilles allograft was transplanted to reconstruct a large Achilles defect (7.0 ± 3 cm)^17^. The patients resumed full weight bearing in 13 wk and achieved single heel raise in 27 wk. In recent case reports, the clinical outcome of Achilles allograft used to repair Achilles defects was promising^18–20^. However, a lack of understanding the biology, biomechanics, and function of Achilles allograft may have limited its application for Achilles reconstruction.

During processing and storage, Achilles allograft is depleted of viable cells. The acellular Achilles allograft has altered mechanical properties and could compromise its healing with host tissues^21^. Repopulation of Achilles allograft with host cells takes at least 12 wk after transplantation^22^. Attempts to overcome the shortcoming of acellularity include to incorporate Achilles allograft with living cells such as tenocytes^23,24^. The healing of a tendon graft with the host tissues, however, involves multiple cell types and takes place in a yet-to-be-defined tissue environment^25^. Mesenchymal stem cells (MSCs) are capable of multilineage differentiation^26^, secrete trophic factors, and suppress inflammation to support tissue regeneration^27,28^. Under the influence of growth differentiation factor-5 (GDF-5), adipose tissue–derived MSCs express genes that are specific for tenocytes^29^. MSCs derived from adipose tissue have been applied to revive a flexor tendon allograft^30^. They were more proliferative and functionally comparable to tenocytes^31^.

This study simulated clinical preparation of Achilles allograft and investigated the applicability of orthotopic transplantation of Achilles allografts and the beneficial effect of adipose tissue–derived MSCs on the revitalization of Achilles allografts in rats. The function of the transplanted Achilles allograft was assessed mechanically, as well as by animal gait and histology.

## Materials and Methods

In this study, 30 Sprague-Dawley rats (Charles River Laboratories, Inc., Wilmington, MA, USA), both male and female at 12 wk of age, were used (approved by MedStar Health Research Institute Institutional Animal Care and Usage Committee).

### Preparation of Achilles Allograft

After the donor rats (*n* = 10) were euthanized, Achilles tendons in both hind limbs were dissected aseptically. The Achilles tendon was harvested in full length, severed at the myotendinous junction proximally, and detached from the calcaneus distally, with a scalpel. The collected tendons (*n* = 20) were trimmed off nontendinous tissues and frozen at −80 °C before transplantation as allografts.

### Isolation of Adipose Tissue–Derived MSCs

Rats (*n* = 20; 10 male and 10 female) were anesthetized with inhalation of 3% isoflurane, and adipose tissue was harvested from both inguinal areas aseptically. Adipose samples were labeled by individual rats and minced. Dissociation of adipose tissue was performed with 1% collagenase (type I; Sigma-Aldrich, St. Louis, MO, USA) in Dulbecco’s modified Eagle’s medium (DMEM; Life Technologies Corp., Carlsbad, CA, USA) at 37 °C for 1 h, with periodical shaking as described previously^32^. After centrifugation at 1,500 rpm for 10 min, the floating fat and supernatant were discarded. The pellet (the cells of the stromal vascular fraction (SVF))) was resuspended in phosphate-buffered saline (PBS) and collected by centrifugation. Cells were counted using a hemocytometer and plated at a density of 3,000 cells/cm^2^. Tissue culture medium was DMEM supplemented with 10% fetal bovine serum (FBS; Rocky Mountain Biologicals, Inc. Missoula, MT, USA) and 1% penicillin-streptomycin (pen-strep) (Life Technologies Corp). Cell culture medium was changed twice a week. The cells were cultured at 37 °C, with 5% carbon dioxide in air and passaged at 75% confluence.

Cell surface markers were analyzed with flow cytometry, using phycoerythrin (PE)-conjugated monoclonal antibodies of CD31, CD34, CD45, CD90 (BD Biosciences, San Jose, CA, USA), and CD44 (GeneTex, Inc., Irvine, CA, USA). PE-conjugated normal mouse IgG1-γ (BD Biosciences) was used as an isotype control. Cells (1 × 10^5^) were suspended in fluorescein-activated cell sorting (FACS) buffer (BD Biosciences) with 2% FBS and incubated with individual antibodies listed above for 1 h at concentrations suggested by the manufacturer. Cells were washed thrice using PBS, resuspended in 250 µL FACS buffer, and analyzed on an Accuri C6 Flow Cytometer (BD Biosciences). Data were analyzed using FCS Express 6 (De Novo Software, Glendale, CA, USA).

A portion of the isolated MSCs were cultured for trilineage differentiation. (1) For osteogenic differentiation, cells were plated in 24-well plates and cultured in DMEM, which was supplemented with 10% FBS, 10 mM β-glycerophosphate, 100 nM dexamethasone, 50 µg/mL l-ascorbic acid 2-phosphate, and 100 ng/mL human recombinant bone morphogenetic protein-2 (BMP-2, Medtronik Sofamer Denek, Memphis, TN, USA). (2) For adipogenic differentiation, MSCs were cultured in DMEM supplemented with 10% FBS, 10 μM rosiglitazone, 1 μM dexamethasone, and 10 ng/mL bovine insulin^33^. (3) Chondrogenic differentiation of MSCs was conducted in pellet culture (3 × 10^5^ cells/pellet). The pellets were cultured in DMEM, which was supplemented with 10% FBS, 50 μ/mL l-ascorbic acid 2-phosphate, 40 μg/mL l-proline, 0.1 μM dexamethasone, and 10 ng/mL recombinant human transforming growth factor β1 (TGF-β) (Peprotech, Rocky Hill, NJ, USA). Recombinant human BMP-7 (100 ng/mL; Peprotech) was added into the chondrogenic media at days 3 and 12 of tissue culture^34^. The controls of trilineage differentiation were the same batch of MSCs cultured in regular growth media. Tissue cultures of osteogenic, adipogenic, and chondrogenic differentiation were maintained for 3 wk and fixed with 4% paraformaldehyde (PFA). The adipogenic cultures were treated with 60% isopropanol, and lipid droplets were stained with Oil Red O (0.3% in polyethylene glycol; Sigma-Aldrich) for 10 min. Matrix mineralization in osteogenic cultures was visualized with Alizarin Red S (2%, pH 4; Sigma-Aldrich) staining, which included incubation for 45 min at room temperature. To analyze chondrogenic differentiation, frozen sections of chondrogenic pellets were stained with Alcian blue (1%; dissolved in 3% acetic acid; pH 2.5; Sigma-Aldrich) for 30 min for extracellular deposition of proteoglycans.

#### Tenogenic differentiation

Before applying onto Achilles allografts, MSCs were treated with human recombinant GDF-5 (100 ng/mL, Peprotech) for 7 d^29^. The differentiation of MSCs was evaluated with quantitative polymerase chain reaction (PCR). Samples of MSCs in tenogenic differentiation cultures and the corresponding controls were lysed with TRIzol (Life Technologies). RNA was extracted by phase separation following the addition of chloroform. First-strand cDNA synthesis was performed using the iScript Reverse Transcription Supermix (Bio-Rad Laboratories, Hercules, CA, USA) with 100 ng of RNA template.

For quantitative gene expression, first-strand cDNA was amplified using the SSO Advanced SYBR Green Supermix (Bio-Rad Laboratories) on a CFX-Connect Real-Time PCR System (Bio-Rad Laboratories). After initial denaturation at 95 °C for 30 s, PCR was performed with 40 cycles consisting of 10 s at 95 °C and 30 s at 53 °C to 59 °C (depending on the primer pair). Standard curves with varying concentrations of cDNA were performed with each primer pair to calculate amplification efficiency. An efficiency of >90% was required for each primer pair and annealing temperature. The specificity of each primer pair was verified using a melting curve from 65 °C to 95 °C and agarose gel electrophoresis of the PCR product. Genes investigated were scleraxis, tenomodulin, and tenascin. The expression of ubiquitin was used as an internal reference. Details of primers (Integrated DNA Technologies, Coralville, IA, USA) are listed in Table 1. Gene expression by the GDF-5-treated MSCs was expressed as fold change from undifferentiated MSCs, using the ΔΔCT method (User Manual, Bio-Rad Laboratories).

**Table 1.** A List of Primers.

Gene Name	Forward Primer Sequence	Reverse Primer Sequence	Product Size (bp)
Ubiquitin	ACACCAAGAAGGTCAAACAGGA	CACCTCCCCATCAAACCCAA	99
Scleraxis	CGAAGTTAGAAGGAGGAGGGT	CGCTCAGATCAGGTCCAAAG	108
Tenascin	GCTACTCCAGACGGTTTC	TTCCACGGCTTATTCCAT	199
Tenomodulin	GGACTTTGAGGAGGATGG	CGCTTGCTTGTCTGGTGC	128

### Orthotopic Transplantation of Achilles Allograft

The rats that donated adipose tissues were anesthetized, and the animals’ left hind limbs were shaved and prepped with alternating alcohol and betadyne. A posterior incision was made over the Achilles tendon, which was subsequently resected from its bony insertion to 1 mm distal to the myotendinous junction.

The animals were randomly selected for receiving an Achilles allograft with or without incubation of autologous MSCs and designated as Allo + MSC group and Allo group, respectively. On the day of surgery, adipose tissue–derived MSCs were trypsinized and collected. After rehydration and thawing, 10 Achilles allografts were incubated with MSCs (1 × 10^5^ in 50 μL PBS per allograft), whose origin matched with the receipt rats, for 30 min. Another 10 Achilles allografts were incubated with PBS (50 μL). Achilles allografts, with or without autologous MSCs, were implanted into the surgically created Achilles defect in the left hind limb of the rats.

Proximally, a modified locking, grasping stitch with absorbable 2-0 Vicryl suture (Ethicon, Somerville, NJ, USA) was utilized to secure the allograft to the gastrocnemius and soleus muscles. Distally, the Achilles allograft was attached to the calcaneus via bone tunnels and sewn back onto itself in a figure-of-eight stitch incorporating the plantaris stump. The wound was closed with a running absorbable 4-0 Vicryl suture. Rats were not restricted from physical activity and allowed to bear weight *ad lib*.

### Gait Analysis

The gait of the rats that received Achilles allograft was recorded with the CatWalk 7.1 gait analysis system (Noldus Co, Leesburg, VA, USA). In the week prior to Achilles allograft transplantation (week 0), rat gait was taken as a baseline. After the surgical procedure, rat gait analysis was performed weekly for 4 wk. Each time, the rat walked through the recording walkway 3 times and at least 5 consecutive steps were included in each walk. Gait recordings of the 3 walks were averaged to minimize unintended gait alterations.

The images of paw prints were processed using the CatWalk program. Parameters that are particularly relevant to Achilles function, such as intensity (brightness of pixels; range = 0 to 255), stance (time in seconds), duty cycle (% = stance/(stance + swing)), were abstracted for analysis.

All rats were euthanized at 4 wk after Achilles allograft transplantation. The transplanted Achilles allografts, with or without MSCs, and the native Achilles tendons in the opposite limbs were harvested for the biomechanical test and histology.

### Biomechanical Test

Achilles transplants, with or without MSCs, and native Achilles tendons from the nonoperated right limbs (*n* = 7 in each group) were used for the loading test. After the rats were sacrificed, the gastrocnemius–soleus complex was dissected free from the femur proximally. Distally, the limb was disarticulated through the hind paw joint preserving the Achilles insertion on the calcaneus. Using a modified Krackow locking suture (# 2 Ethibond suture; Ethicon Inc.), the myotendinous junction proximal to the allograft was secured and the sutures were anchored to a grip site in the biomechanical testing frame (858 Mini Bionix, MTS Systems Corp., Eden Prairie, MN, USA). Distally, the dissected hind limb was anchored to the frame via 2 Kirschner wires, which passed through the calcaneus transversely to prevent the foot from rotating. The construct was preloaded, and tensile forces until ultimate failure were recorded.

### Histology

Tissue samples of Achilles transplants, with or without MSCs, and native Achilles tendons in the nonoperated limbs were divided into 3 portions for histological analysis: (1) the proximal portion included the junction of Achilles allograft and host gastrocnemius–soleus complex, (2) the middle portion included the substance portion of the Achilles allograft, and (3) the distal portion included the attachment of distal Achilles allograft to calcaneus. The tissue blocks were embedded in Tissue-Tek® O.C.T. Compound (Sakura Finetek USA, Torrance, CA, USA) and sectioned along the long axis of the tendon with a cryostat (CM 1950; Leica Biosystems, Buffalo Grove, IL, USA). Randomly selected tissue sections were stained with hematoxylin and eosin (H&E) and Picrosirius Red separately. Images of H&E staining were taken at the center of Achilles substance sections. On each image, in a computer-defined area, cellularity of Achilles tendon was graded as 1 = existence of an acellular area, 2 = sparse cell distribution, and 3 = dense cell distribution.

Images of Picrosirius Red staining were taken under a polarizing microscope (Olympus BH-2, Olympus Corp., Tokyo, Japan). To analyze collagen composition, the images were split into red, green, and blue channels (ImageJ, National Institutes of Health, Bethesda, MD, USA)^35^. The mean intensity in the histograms of red and green channels of the images was recorded. The ratio of the staining intensity of the fine (green) collagen fibers and thick (red) collagen fibers in Achilles transplants and native Achilles tendons was calculated for comparison.

#### Statistical analysis

The expression of genes related to tenogenesis was compared between the MSCs treated with or without GDF-5, using *t* test. Gait parameters collected postsurgery were compared with their baselines at week 0, for gait features of Allo and Allo + MSC groups, and between the operated and nonoperated limbs in the same group for the effect of grafting, using 2-way analysis of variance (ANOVA), followed by *post hoc* Fisher test. Gait parameters at the end point (week 4) were normalized to week 0 as the percentage of recovery and compared between the Allo and Allo + MSC groups with Mann–Whitney *U* test. Mechanical strength of Achilles tendons in the Allo, Allo + MSC, and native Achilles tendon groups and deposition of fine collagen fibers in the proximal, middle, and distal portions of the allograft were analyzed using 1-way ANOVA, followed by *post hoc* Fisher test. The histological scores of cellularity were compared between the Allo and Allo + MSC groups with Mann–Whitney *U* rank test. Statistical analyses were performed using Minitab 17 (Minitab Inc., State College, PA, USA). Significance was set as *P* < 0.05.

## Results

### Characterization of MSCs

**Fig. 1.**
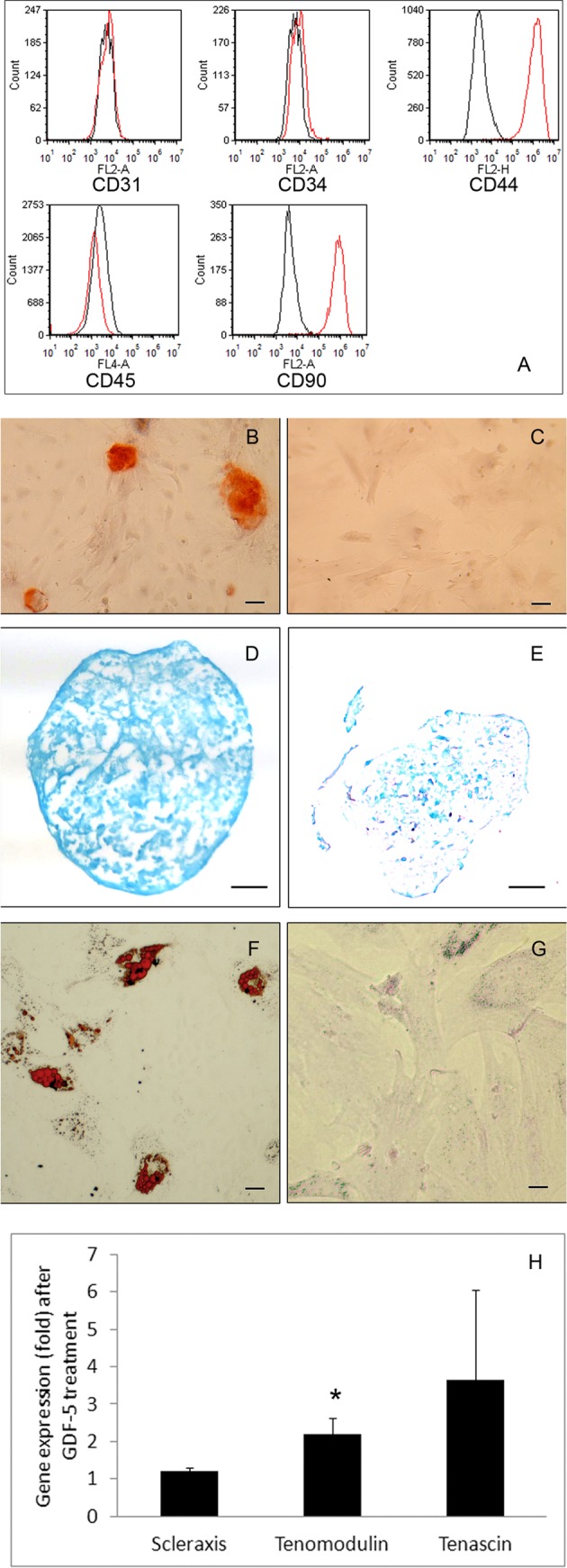
Characterization of adipose tissue–derived mesenchymal stem cells (MSCs). A panel of cell surface markers examined by **Fig. 1.** (continued). flow cytometry (A). The adipose tissue–derived MSCs do not express CD31, CD34, and CD45 but express CD44 and CD90. In osteogenic cultures, there is mineralization in the extracellular matrix (red; B) but not in the control cultures (C; Alizarin Red staining; bar = 50 µm). Chondrogenic pellet cultures show abundant proteoglycans (blue; D), but the control pellet (E) has little proteoglycans in the matrix (Alcian Blue staining; bar = 100 µm). In adipogenic cultures, fat droplets are developed intracellularly (red; F) but not in the control cultures (G; Oil Red O staining; bar = 20 µm). The expression of scleraxis and tenascin by MSCs treated with or without growth differentiation factor-5 (GDF-5) is not different statistically (H). The expression of tenomodulin by MSCs treated with GDF-5, however, is significantly increased over the control (*P* < 0.05).

The adipose tissue–derived MSCs were negative for CD31, CD34 and CD45, but positive for CD44 (97% ± 1%) and CD90 (98% ± 1%; Fig. 1A). After being cultured in adipogenic, chondrogenic, and osteogenic media, MSCs developed fat droplets in the cellular plasma, deposited proteoglycans in the extracellular matrix, and mineralized extracellular matrix, respectively (Fig. 1B to G). After treatment with GDF-5, MSCs had increased expression of tenomodulin (about 2.2-fold (*P* < 0.05; Fig. 1H)). The expression of scleraxis and tenascin was not statistically different from the control cultures.

### Orthotopic Transplantation of Achilles Allograft with or without Autologous MSCs

After Achilles allograft transplantation, rats in both Allo and Allo + MSC groups were able to use the operated limbs to stand and walk immediately. All animals survived the surgeries of harvesting fat tissue and transplantation of Achilles allograft. No wound infection developed in the operated rats.

### Gait Analysis

**Fig. 2.**
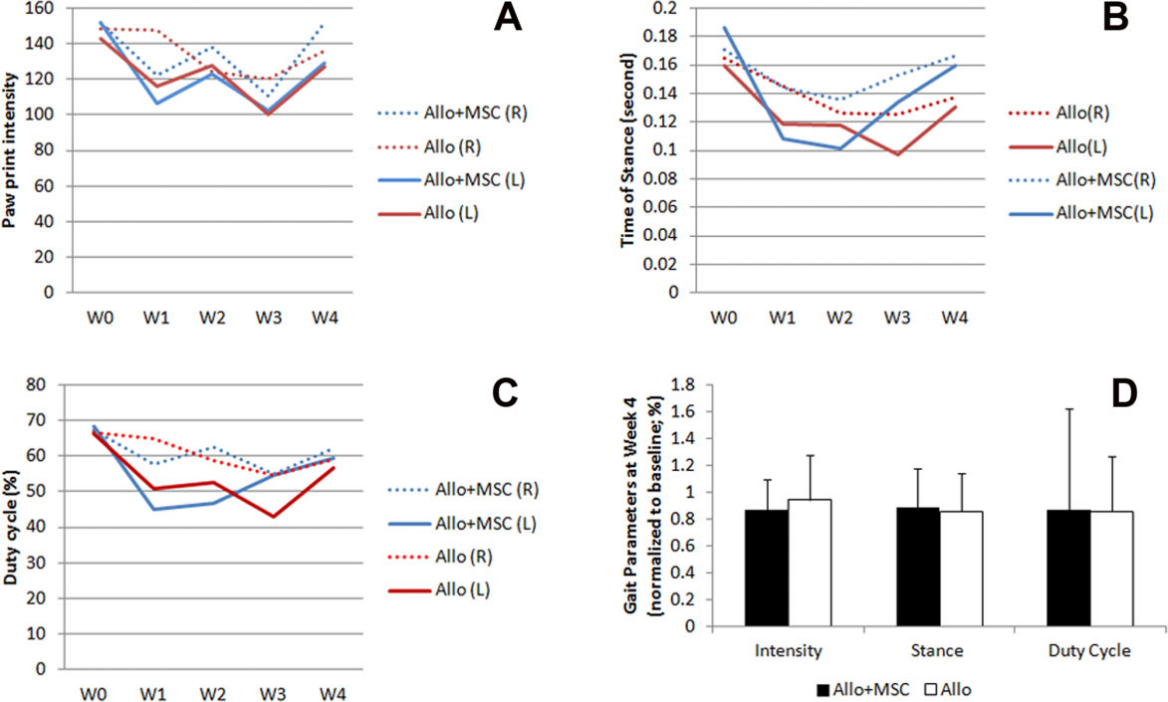
Gait analysis of rats received Achilles allograft. (A) Paw intensity of the operated limbs in Allo + mesenchymal stem cell (MSC) group (blue, solid line) and Allo group (red, solid line) is reduced from week 1 through week 3. At week 4, paw intensity of the operated left limbs in both groups is largely recovered and not different between the 2 groups. The paw intensity of the nonoperated limbs is shown as dotted line (red, Allo group; blue, Allo + MSC group). (B) The stance time of the operated limbs in both Allo and Allo + MSC groups is reduced at week 1. At week 4, the stance time of the operated left limbs in both groups is similar to the nonoperated limbs. (C) The duty cycle is reduced in the operated limbs in both Allo and Allo + MSC groups at week 1. In Allo + MSC group, the operated and nonoperated limbs share similar duty cycle at weeks 3 and 4. In Allo group, it is until week 4 that the 2 limbs become similar in duty cycle. (D) The recovery of paw intensity, stance, and duty cycle, in percentage over week 0, of the operated limbs at week 4 is comparable between Allo and Allo + MSC groups.

The print intensity of the operated limbs fluctuated during the 4-wk follow-up, and the Allo and Allo + MSC groups shared a similar pattern of paw print intensity. The operated (left) limbs reduced paw print intensity about 20% from baseline in the Allo group (*P* < 0.05) and 30% in the Allo + MSC group in week 1 (*P* < 0.05; Fig. 2A). By week 4, the print intensity in the Allo and Allo + MSC groups were about 89% and 85% of their respective baseline (*P* > 0.05 for both groups; Fig. 2D). The print intensity in the opposite (nonoperated, right) limb in both groups reached or nearly reached the corresponding baselines in week 4. In both groups, the paw intensity between the operated and nonoperated limbs was not significantly different at week 4 (*P* > 0.05).

At week 1, the stance time of the limbs that received Achilles allograft was reduced from week 0 in both groups, with the Allo + MSC group to a greater degree (*P* < 0.05; Fig. 2B). This condition lasted through weeks 2 and 3. By week 4, the stance time in both groups largely recovered (Allo group = 85% and Allo + MSC group = 88%) and became virtually indistinguishable from their respective baseline at week 0 (*P* > 0.05). At week 4, the stance time between Allo and Allo + MSC groups was not significantly different (*P* > 0.05; Fig. 2D). In both groups, the stance time of the operated limb and nonoperated limb was not different at week 4 (*P* > 0.05).

The duty cycle (percentage of the stance phase in a step cycle) of the reconstructed limbs in both Allo and Allo + MSC groups was reduced, compared with its baseline, through weeks 1 to 4 (*P* < 0.05 in both groups; Fig. 2C). While it was gradually recovering after week 2 in the Allo + MSC group, the recovery did not start until week 4 in the Allo group. At week 4, the duty cycle improved to about 85% and 88% of its baseline in the Allo group and the Allo + MSC group, respectively. The recovery rate of the duty cycle (in percentage over the baseline) between the Allo and Allo + MSC groups was not statistically different (Fig. 2D).

**Fig. 3.**
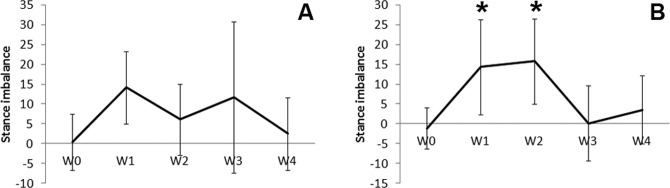
Stance balance calculated based on duty cycle. In the Allo group, stance imbalance between the operated and nonoperated limbs shows from week 1 to 3 but is not statistically significant (A; W = week). In the Allo + mesenchymal stem cell (MSC) group, there is significant stance imbalance at week 1 and 2. The imbalance diminishes at week 3 and 4 (B; W = week).

Imbalance of stance was calculated as the difference of duty cycle between the nonoperated limb and operated limb. While the imbalance of stance was not significant in the Allo group, it was significantly increased in the Allo + MSC group in weeks 1 and 2 as compared with week 0 (*P* < 0.05; Fig. 3). The imbalance of stance in the Allo + MSC group diminished in weeks 3 and 4.

### Biomechanical Testing

During the loading test, all the samples were ruptured at the proximal myo-tendineous junction. Maximum load of failure of Achilles allograft/tendon was not significantly different among the Allo group, Allo + MSC group, and native Achilles tendon group (*P* > 0.05; Table 2). The stiffness of the allograft, with or without MSCs, and native Achilles tendons was not different statistically (*P* > 0.05).

**Table 2.** The Mechanical Properties of Achilles Allograft and Native Achilles Tendon.

	Max Load (*N*)	Stiffness (*N*/mm)
Allo	27.2 ± 11.5	2.4 ± 0.9
Allo + MSC	27.6 ± 6.4	2.2 ± 1.3
Native	19.9 ± 9.9	3.1 ± 2.0

Abbreviations: Max load, maximum load of failure; MSC, mesenchymal stem cell.

### Histological Analysis

**Fig. 4.**
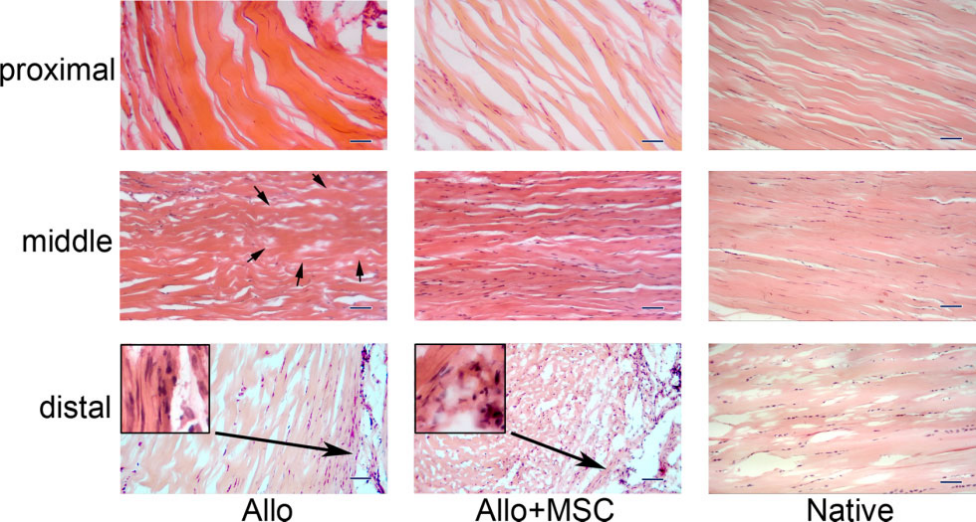
Histology of the transplanted Achilles allograft, with or without mesenchymal stem cells (MSCs). For both Allo and Allo + MSC groups, there are fibroblasts invaded in the proximal portion of the allograft, and some of the cells appear elongated and tenocyte-like. In the middle segment of the allograft, while acellular area (outlined with arrows) exists in the Allo group, the allograft is fully repopulated in the Allo + MSC group. There are abundant fibroblasts that infiltrated into the distal portion of the allograft in both groups, particularly at the peripheral region (inserts in higher magnifications). The corresponding proximal, middle, and distal portions of native Achilles tendon are posted for control (hematoxylin and eosin staining; bar = 100 µm).

**Fig. 5.**
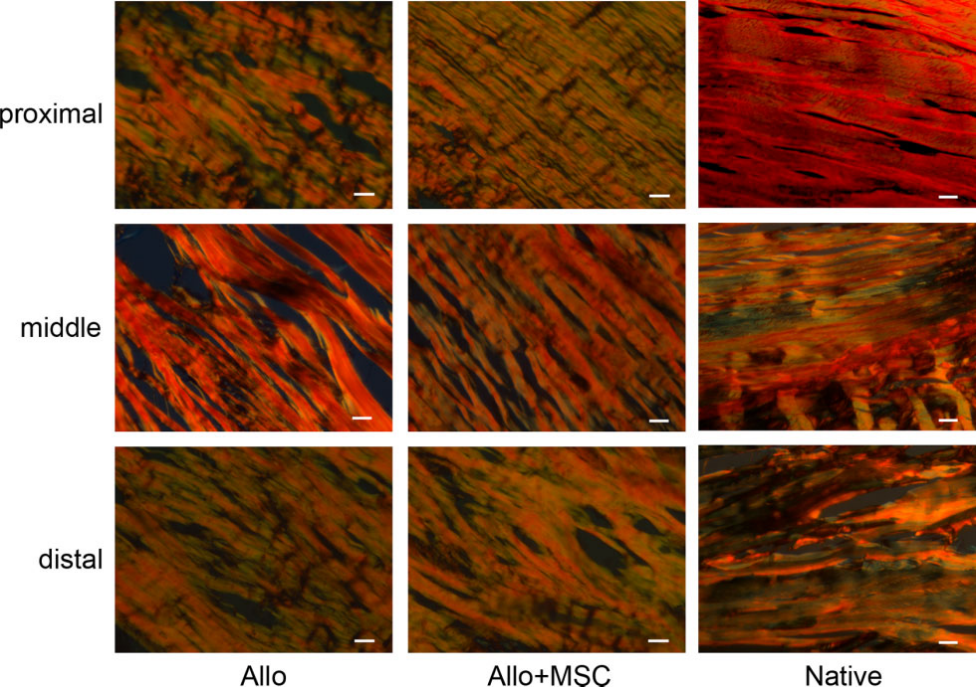
Collagen composition in Achilles allograft by polarizing microscopy. The allograft in both Allo and Allo + mesenchymal stem cell (MSC) groups and native tendons are overwhelmingly stained for thick (type I) collagen fibers (red) in all 3 segments. Compared with the native Achilles tendon, the proximal and distal portions of the allograft in both Allo and Allo + MSC groups have increased deposition of fine (type III) collagen fibers (green). In the middle portion of the allograft, while the deposition of fine collagen fibers mostly appear around the large collagen bundles in the Allo group, it takes place inside of the collagen bundles in the Allo + MSC group (picrosirius Red staining; bar = 50 µm).

**Fig. 6.**
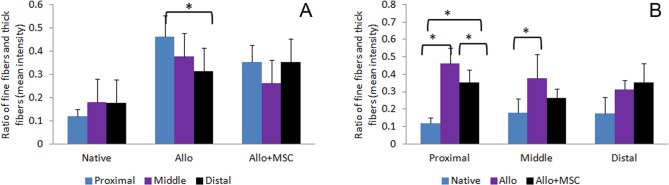
The ratio of fine (type III) collagen fibers and thick (type I) collagen in native Achilles tendons and allografts, based on picrosirius Red staining. In the Allo group, the ratio of fine/thick collagen fibers is increased in the proximal section, compared with the distal portion (A). The ratio, however, is consistent in the proximal, middle, and distal sections in the native Achilles tendons and the Allo + mesenchymal stem cell (MSC) group. The ratio of fine/thick collagen fibers in the proximal sections is significantly different among the native Achilles tendons, the Allo group, and the Allo + MSC group (B). It is the greatest in the Allo group. For the middle section, the ratio is increased in the Allo group as compared with the native Achilles tendons. The ratio in the distal sections is not significantly different among the 3 groups. *P < 0.05.

On histology, Achilles allograft healed with gastrocnemius and soleus muscles proximally and calcaneus distally in both Allo and Allo + MSC groups (Fig. 4). Around the allograft, particularly in the distal section, there were significant amounts of fibroblasts penetrating into the allograft. While hypercellularity presented in the allograft peripherally, acellularity persisted in the central area of the allograft, particularly in the Allo group. The cellularity of Achilles allograft was higher in the Allo + MSC group than in the Allo group (average cellularity grade 2.7 ± 0.5 vs. 1.7 ± 0.5; *P* < 0.05). Under a polarizing microscope, the proximal and distal portions of Achilles allograft in both Allo and Allo + MSC groups had similar patterns of distribution and composition of thick and fine collagen fibers (Fig. 5). Fine collagen fibers (green) were deposited around, and inside, the fiber bundles which were dominated with thick collagen fibers (red). In the middle portion of the Achilles allograft, deposition of fine collagen fibers was mostly around the collagen bundles in the Allo group. In the Allo + MSC group, they were also deposited inside the fiber bundles. Analysis of the polarizing images revealed that, compared with the native Achilles tendons, there was an increased ratio of fine/thick collagen fibers in the proximal, middle, and distal portions of the allograft in both Allo and Allo + MSC groups (range from 30% to 50%; Fig. 6A). While the ratio of fine/thick collagen fibers was consistent across the proximal, middle, and distal sections in the native Achilles tendons (12% to 18%) and in the Allo + MSC group (26% to 35%), it was significantly increased in the proximal section (46%), as compared with the distal section (31%), in the Allo group (*P* < 0.05). When the native Achilles tendons, Allo group, and Allo + MSC group were compared section by section, the ratio of fine/thick collagen fibers was the greatest in the Allo group (*P* < 0.05; Fig. 6B). Compared with the native Achilles tendons, the ratio of fine/thick collagen fibers in the Allo group was significantly increased (*P* < 0.05). The ratio of fine/thick collagen fibers in the distal section was not significantly different between the native Achilles tendons and the allografts in the Allo and Allo + MSC groups.

## Discussion

This study followed the common procedures of Achilles allograft harvesting and preparation in tissue banks, except radiation for sterilization^36^. The suture technique applied in the current animal model adequately secured the full-length Achilles allograft to the gastrocnemius–soleus complex proximally and the calcaneus distally, as the limbs were able to bear weight immediately postoperation. Orthotopic transplantation of Achilles allograft changed the temporal features and kinematics of rat gait. Paw print intensity reflexes the weight loaded and forces applied on the limb, where a functional Achilles tendon is essential. In the (left) limb, which received Achilles allograft, paw intensity was steadily decreased postsurgery and reduced about a third by week 3. This could be attributable to pain associated with surgical trauma as well as functional deficiency of the transplanted Achilles allograft. By week 4, however, the paw intensity of the operated limb nearly fully recovered to its preoperation baseline. Stance time and duty cycle in the limb that received Achilles allograft were similarly decreased in the first 3 wk and largely reversed in week 4. The temporal balance of the gait, depicted by the imbalance of duty cycle between the operated (left) and nonoperated (right) hind limbs, in the Allo + MSC group was temporarily lost in the first 2 wk after surgery and resumed in weeks 3 and 4. It is possible that this was related to the activities of, or reactions to, the supplemented MSCs because there was no gait imbalance at this period in the Allo group. Nevertheless, gait was balanced in both Allo and Allo + MSC groups at week 4. Taken together, orthotopic transplantation of Achilles allograft briefly affected the gait, particularly when MSCs were implemented, and Achilles allograft was largely functional after 4 wk.

Healing of the implanted Achilles allograft with the gastrocnemius and soleus muscles proximally and calcaneus distally was demonstrated histologically in both Allo and Allo + MSC groups. Mechanical strength and stiffness of the implanted Achilles allograft in 4 wk were comparable to native Achilles tendons. The limb function and biomechanical and histological results of the current study suggest that Achilles allograft can reliably reconstruct Achilles tendon, as it has been for reconstruction and augmentation of other tissues and joints^12,16^.

In this study, adipose tissue–derived MSCs were incorporated onto the Achilles allograft for accelerating revitalization. Adipose tissue was chosen as the source of MSCs because of its abundance and convenience of collection. Taking clinical practice into consideration, MSCs were applied in an autologous fashion.

Undifferentiated MSCs have been cultured with tendon grafts to improve cellularity^37^. Unlike the classic chondrogenic, osteogenic, and adipogenic differentiation, tenogenic differentiation of MSCs is not well defined in terms of conditions and evaluation criteria. Some studies, however, showed that GDF-5 promotes MSCs to acquire certain features of tenocytes, through reorganization of cytoskeleton^29,38^. In this study, GDF-5 treatment indeed increased the expression of tenomodulin, a marker of tenocytes, by MSCs. By upregulation of tenomodulin alone, however, the degree of differentiation of the MSCs toward the tenogenic lineage could not be determined. Incubation of MSCs nevertheless increased the cellularity of Achilles allografts, especially in the middle portion, where acellular areas still existed in the Allo group. The thick and fine collagen fibers defined by polarizing microscopy roughly correspond to types I and III collagen fibers^34^. Compared with native Achilles tendons, an increased content of type III collagen in both Allo and Allo + MSC groups is a sign of tendon repair and remodeling^39^. Detailed sectional analysis showed that deposition of type III collagen in the Allo group varied between the proximal and distal portions. Type III collagen was more evenly distributed across the entire allograft in the Allo + MSC group, which is a pattern shared by the native Achilles tendons. Therefore, the implemented MSCs could have directly or indirectly improved the cellularity and impacted matrix remodeling in the Achilles allograft.

Animals in the Allo + MSC group, but not the Allo group, developed significant stance imbalance in weeks 1 and 2. Stance imbalance is a way to determine limb injury in rodents^40^. In conjunction with reduced paw intensity, abnormal gait is indicative of painful gait^41^. The first 2 wk of tendon transplantation coincide with intense inflammation and wound healing, whereas biological activities relevant to tendon healing mainly occur 2 wk later^25^. The interference of wound healing by the implanted MSCs is unexpected and rarely mentioned in similar procedures. Overall, wound healing in both Allo and Allo + MSC groups was not problematic. At the end of 4 wk, the Allo + MSC group was not different from the Allo group in maximum load of failure, stiffness, and gait.

In this study, supplementation of MSCs increased the cellularity and improved the homogeny of matrix remodeling in Achilles allograft. These effects, however, were not detected by gait analysis and mechanical tests, probably due to limitations of the current animal model.

This study followed the animals for only 4 wk. It took 12 to 30 wk to repopulate the forepaw flexor tendon grafts in rabbits^42^. Orthotopic transplantation of Achilles allograft in a large animal model, with longer follow up, may be a better simulation of the human condition. Another limitation of this study is that there was no tracking of the MSCs in the Allo + MSC group *in vivo*, which would be valuable for determining the fate and role of MSCs in Achilles allografts.

In conclusion, orthotopic transplantation of Achilles allografts largely restored the function of the native tendon. Supplementation of MSCs improved the cellularity and influenced the matrix remodeling in the allografts. Orthotopic transplantation of Achilles allograft, therefore, could be a practical approach to reconstruct/replace the injured or diseased Achilles tendon.
